# Molecular Architecture and Biomedical Leads of Terpenes from Red Sea Marine Invertebrates

**DOI:** 10.3390/md13053154

**Published:** 2015-05-20

**Authors:** Mohamed Elamir F. Hegazy, Tarik A. Mohamed, Montaser A. Alhammady, Alaa M. Shaheen, Eman H. Reda, Abdelsamed I. Elshamy, Mina Aziz, Paul W. Paré

**Affiliations:** 1 Chemistry of Medicinal Plants Department, Center of Excellence for Advanced Sciences, National Research Centre, 33 El Bohouth st., Dokki, Giza, P.O. Box 12622, Egypt; E-Mails: tarik.nrc83@yahoo.com (T.A.M.); alaasaidalah_shaheen@yahoo.com (A.M.S.); dremanhusseinreda@gmail.com (E.H.R.); 2 National Institute of Oceanography and Fisheries, Red Sea Branch, Hurghada 84511, Egypt; E-Mail: coralreef_niof1@yahoo.com; 3 Natural Compounds Chemistry Department, National Research Centre, 33 El Bohouth st. (former El Tahrir st.) Dokki, Giza, P.O. Box 12622, Egypt; E-Mail: elshamynrc@yahoo.com; 4 Department of Chemistry and Biochemistry, Texas Tech University, Lubbock, TX 79409, USA; E-Mail: drmina_aziz06@yahoo.com

**Keywords:** terpenes, Red Sea, marine ecosystem, marine invertebrates, biomedical leads

## Abstract

Marine invertebrates including sponges, soft coral, tunicates, mollusks and bryozoan have proved to be a prolific source of bioactive natural products. Among marine-derived metabolites, terpenoids have provided a vast array of molecular architectures. These isoprenoid-derived metabolites also exhibit highly specialized biological activities ranging from nerve regeneration to blood-sugar regulation. As a result, intense research activity has been devoted to characterizing invertebrate terpenes from both a chemical and biological standpoint. This review focuses on the chemistry and biology of terpene metabolites isolated from the Red Sea ecosystem, a unique marine biome with one of the highest levels of biodiversity and specifically rich in invertebrate species.

## 1. Red Sea Ecosystem

Marine ecosystems cover nearly 70% of the earth’s surface, averaging almost 4 km in depth and are proposed to contain over 80% of the world’s plant and animal species [1]. Exact marine biodiversity is less certain since between one-third and two-thirds of marine organisms have yet to be described [2]. Worldwide there are approximately 226,000 marine eukaryotes currently reported, while close to a million total species are estimated, based on calculations by marine biologists using statistical predictions [2]. Considering that constituents from higher plants along with metabolites from terrestrial microorganisms have provided a substantial fraction of the natural-product-derived drugs currently used in Western medicine [3], the potential to vastly expand the number and diversity of natural products by mining marine eukaryotes as well as associated prokaryotes from the richly diverse Red Sea ecosystem seems attainable. In fact, just within the past quarter century, the search for new marine metabolites has resulted in the isolation of upward of 10,000 compounds [4], many of which exhibit biological activity. Despite the fact that marine biodiversity far exceeds that of terrestrial ecosystems, research of marine natural products as pharmaceutical agents, is still in its infancy. Factors that contribute to the gap between terrestrial and marine derived natural products include a paucity of ethno-medical history from marine sources as well as impediments associated with collecting, identifying and chemical analysis of marine materials [5].

Notwithstanding, a combination of new diving techniques and implementation of remotely operated pods over the last decade has facilitated the characterization of marine-derived metabolites. This review encompasses secondary metabolites derived from marine invertebrates, a largely diverse group of fixed or sessile organisms, many in a stationary form although some are capable of slow primitive movement. While invertebrates lack physical defences such as a protective shell or spines, they are often rich in defence metabolites that can be utilized to attack prey or defend a habitat.

**Figure 1:**
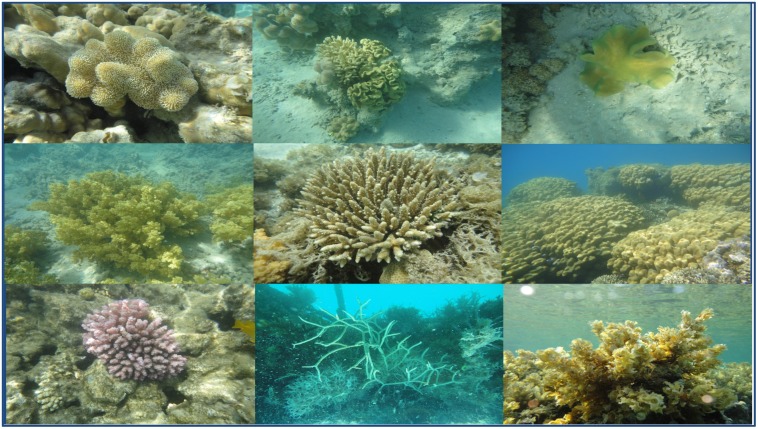
Samples of marine invertebrate diversity from the Red Sea including (from left to right starting at the top left corner) *Sarcophyton glaucum*, *S. regulare*, *S. ehrenbergi*, *Nephthea molle*, *Acropora humilis*, *Porites solida*, *Pocillopora verrucosa*, *Clothraria rubrinoidis* and *Cystoseira trinode*. Marine species exhibit greater phyta diversity than land species.

**Figure 2:**
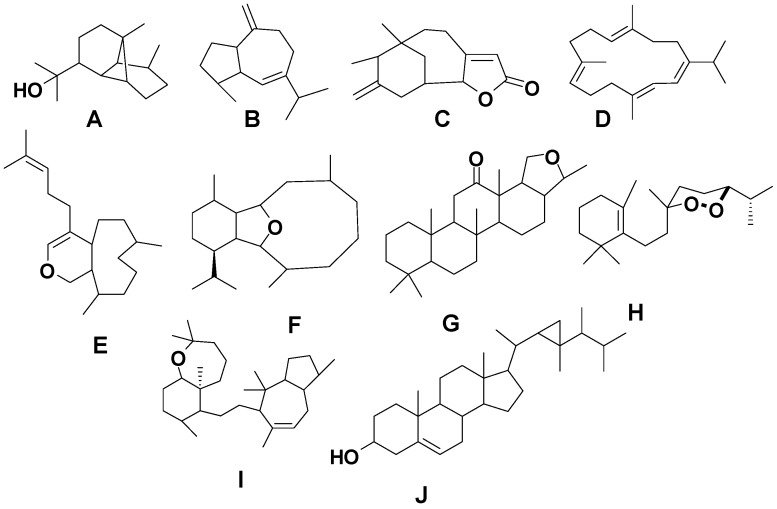
Terpene skeletal types including ylangene (A), aromadendrane (B), tricycle-[6,7,5]-sesquiterpene (C), cembrane (D), xeniolide and xeniaphyllane (E), eunicellin diterpene (F), sesterterpene (G), norsesterterpene (H), triterpene (I) and steroid (J) types.

This review focused on a class of secondary defence metabolites abundant in marine invertebrates, five-carbon isoprenoid-derived terpenes. Extensive speciation from microorganisms to mammals can be attributed, at least in part to a wide range of temperatures (0 to 35 °C in arctic waters versus hydrothermal vents, respectively), pressures (1–1000 atm.), nutrient availabilities (oligotrophic to eutrophic) and lighting conditions that exist in this marine biome [6]. The analysis will be limited to the Red Sea which is considered an epicenter for marine biodiversity with an extremely high endemic biota including over 50 genera of hermatypic coral. Indeed soft coral (Cnidaria: Anthozoa: Octocorallia), which are an important structural component of coral reef communities [7,8], are approximately 40% native to the Red Sea [6]. The Red Sea, in which extensive reef formation occurs, is arguably the world’s warmest (up to 35 °C in summer) and most saline habitat (*ca*. 40 psu in the northern Red Sea) [6]. Despite the Red Sea’s size and diversity of reef-associated inhabitants (for examples, see Figure 1), marine invertebrates in this ecosystem remain poorly studied compared to other large coral reef systems around the world such as the Great Barrier Reef or the Caribbean. This review will cover terpenes isolated from marine invertebrates of the Red Sea (Figure 2) as well as identified biological activities for compounds reported during the time period from 1980 to 2014.

## 2. Sesquiterpenes

Sesquiterpenes are secondary metabolites present in many marine organisms including soft coral (*e.g*., *Dendronephthya* sp., *Sinularia gardineri*, *Litophyton arboreum*, *Sarcophyton trocheliophorum*, *S. glaucum* and *Parerythropodium fulvum fulvum*) [9,10,11,12,13,14], and sponges (*e.g*., *Hyrtios* sp. and *Diacarnus erythraenus*) [15,16].

### 2.1. Ylangene-Type Sesquiterpenes

**Figure 3:**
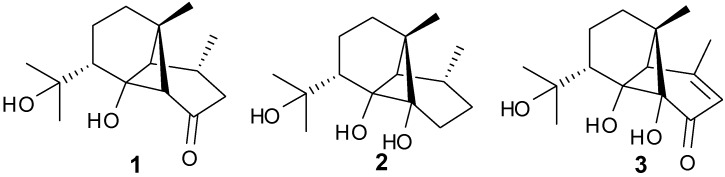
Representative structures of ylangene-type sesquiterpenes (**1**–**3**).

Tricyclo-[4,6,6]-sesquiterpenes, Dendronephthol A–C (**1**–**3**) have been isolated from the soft coral *Dendronephthya*, family *Nephtheidae* (Figure 3). Cytotoxic activity was observed for **1** and **3** against the murine lymphoma L5187Y cancer cell line with ED_50_ values of 8.4 and 6.8 μg/mL, respectively [9].

### 2.2. Aromadendrane-Type Sesquiterpenes

**Figure 4:**
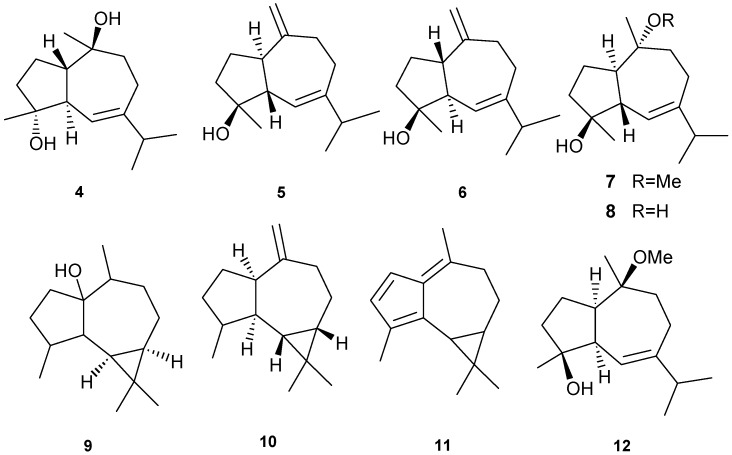
Representative structures of aromadendrane-type sesquiterpenes (**4**–**12**).

Bicyclico [5,7] sesquiterpenes have been isolated from several different coral with examples shown in Table 1 and Figure 4. Cytotoxicity to murine leukemia (P-388), human lung carcinoma (A-549), human colon carcinoma (HT-29), and human melanoma cells (MEL-28) [11] was observed with exposure to **4**. Inhibitory activity against HIV-1 protease (PR) at an IC_50_ of 7 μM was observed for **5**. Compounds **5** and **8** demonstrated cytostatic action with assaying HeLa cells, revealing potential use in virostatic cocktails [11]. Antitumor activity against lymphoma and Ehrlich cell lines was observed for **9** with LD_50_ in the range of 2.5–3.8 μM; antibacterial and antifungal activities were also observed [12]. Compound **10** showed potent activity against the prostate cancer line PC-3 with an IC_50_ of 9.3 ± 0.2 μM. Anti-proliferative activity of **9** can be attributed, at least in part, to its ability to induce cellular apoptosis [13]. Compound **12** exhibited a promising IC_50_ > 1 μg/mL against three cancer cell lines including murine leukemia (P-388; ATCC: CCL-46), human lung carcinoma (A-549; ATCC: CCL-8) and human colon carcinoma (HT-29; ATCC: HTB-38) [15].

**Table 1:** Aromadendrane sesquiterpenes, sources and activities.

No.	Name	Sources	Activities
4	Guaianediol [10]	*Sinularia gardineri*	anti-tumor
5	Alismol [11]	*Litophyton arboreum*	cytostatic
6	Lactiflorenol [17]	*Sinularia polydactyla*	
7	10-*O*-Methyl alismoxide [11]	*L. arboreum*	
8	Alismoxide [11]	*L. arboreum*	cytostatic
9	Palustrol [12]	*Sarcophyton trocheliophorum*	anti-tumor, antibacterial and antifungal
10	10(14)-Aromadendrene [13]	*Sarcophyton glaucum*	anti-tumor, antiproliferative
11	Fulfulvene [14]	*Parerythropodium fulvum fulvum*	
12	*O*-Methyl guaianediol [15]	*Diacarnus erythraenus*	cytotoxic

### 2.3. γ-Methoxybutenolide-Type Sesquiterpenes

**Figure 5:**
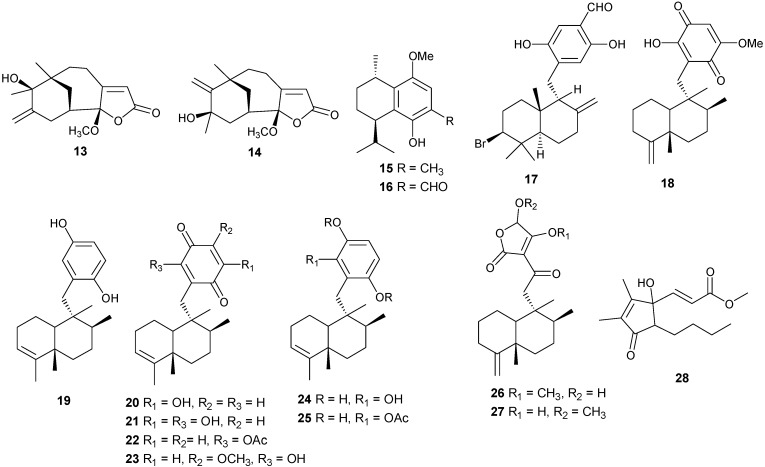
Structures of sesquiterpenes-γ-methoxybutenolides and sesquiterpene derivatives (**13**–**28**).

Tricyclo-[6,7,5]-sesquiterpenes, Hyrtiosenolide A and B have been isolated from the sponge *Hyrtios* sp., and compounds **13** and **14** exhibit weak antibacterial activity against *Escherichia coli* [16] (Figure 5).

### 2.4. Miscellaneous Sesquiterpenes

Additional sesquiterpenes have been isolated from several coral genera with examples reported in Table 2 and Figure 5. Compound **28** exhibits cytotoxic activity against human hepatocarcinoma (HepG2) and breast adenocarcinoma (MCF-7) [17].

**Table 2:** Other sesquiterpenes, sources and activities.

No.	Name	Sources	Activities
15	5-Hydroxy-8-methoxy-calamenene [14]	*Parerythropodium fulvum fulvum*	
16	5-Hydroxy-8-methoxy-calamenene-6-al [14]	*Parerythropodium fulvum fulvum*	
17	Peyssonol A [17]	*Peyssonnelia* sp.	
18	Ilimaquinone [18]	*Smenospongia* sp.	
19	Avarol [18,19]	*Dysidea cinerea*	HIV
20	3′-Hydroxyavarone [20]	*D.cinerea*	
21	3′,6′-Dihydroxyavarone [20]	*D.cinerea*	
22	6′-Acetoxyavarone [20]	*D.cinerea*	
23	6′- Hydroxy4′-methoxyavarone [20]	*D.cinerea*	
24	6′-Hydroxyavarol [20]	*D.cinerea*	
25	6′-Acetoxyavarol [20]	*D.cinerea*	
26	Smenotronic acid [18]	*Smenospongia* sp.	
27	Dactyltronic acids [18]	*Smenospongia* sp.	
28	(*E*)-Methyl-3-(5-butyl-1-hydroxy-2,3-dimethyl-4-oxocyclopent-2-enyl)acrylate [21]	*Sarcophyton ehrenbergi*	cytotoxic (HepG2) (anti-tumor)

## 3. Diterpenes

Diterpenoids are widespread in various marine organisms including soft coral (*Sarcophyton glaucum*, *S. trocheliophorum*, *Sinularia polydactyla*, *S. gardineri*, *Litophyton arboreum*, *Lobophyton* sp., *Xenia* sp. and *Cladiella pachyclados*) [10,11,12,13,22,23,24,25,26,27,28,29], and sponges (*Leucetta chagosensis*) [23].

### 3.1. Cembrane-Based Diterpenes

**Figure 6:**
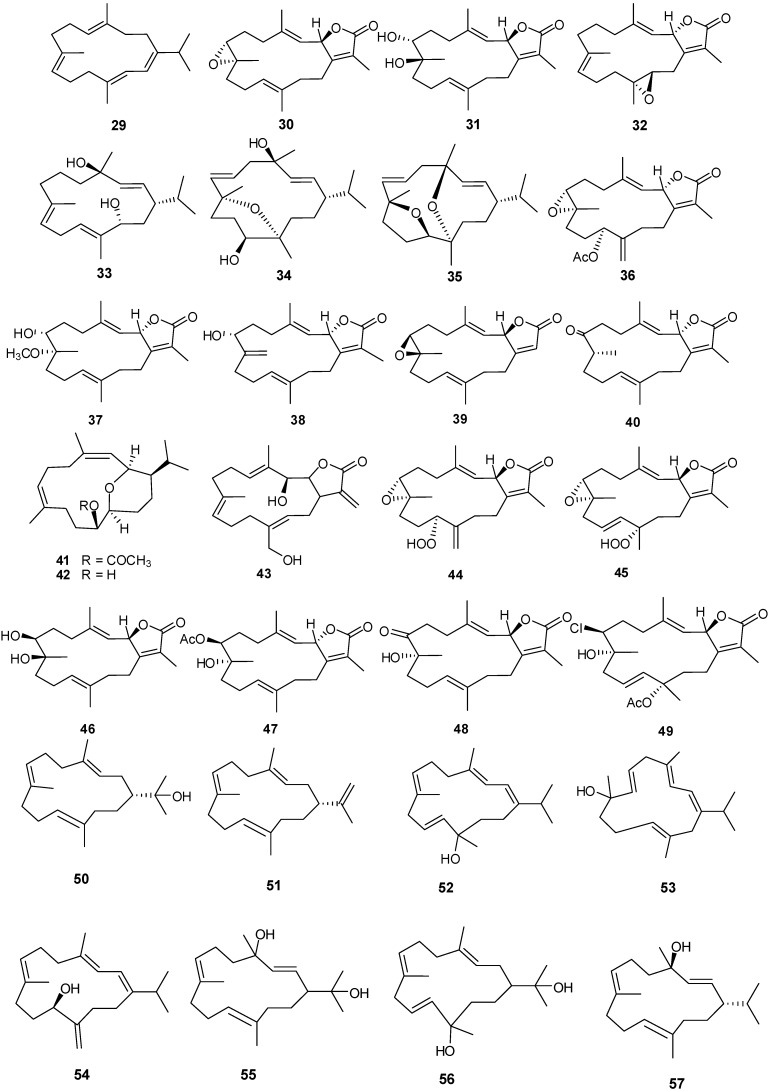

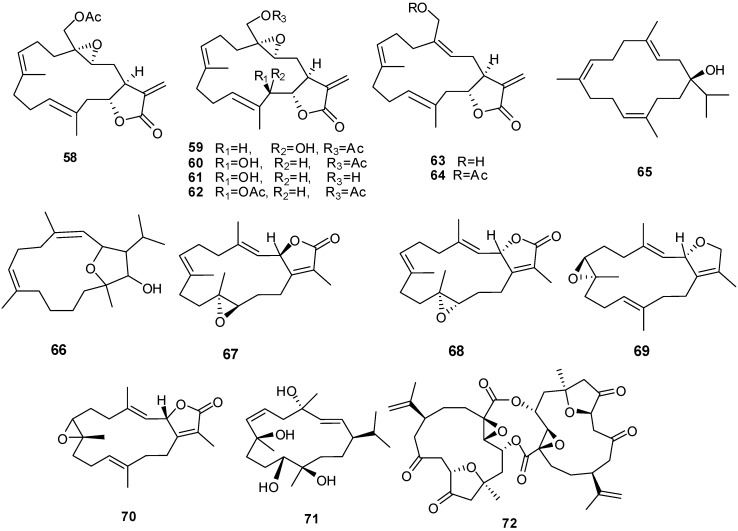
Structures of cembrane-based diterpenes (**29**–**72**).

Fourteen-membered cyclic and bicycle-[5,14]-diterpenes have been isolated from numerous coral genera with examples shown in Table 3 and Figure 6. Compounds **29**, **41** and **42** exhibited antibacterial and antifungal activity against *Aspergillus flavus* and *Candida albicans* with low μM MIC values [12]. Lack of cytotoxicity against monkey kidney CV-1 cells suggests that **30**, **32**, and **33** may prove to be good candidate drugs against melanoma and warrant further studies in the development as antitumor agents [19]. Compound **30** exhibits moderate antifungal activity against *Cryptococcus neoformans* with an IC_50_ of 20 μg/mL [22]. Compound **43** showed selective cytotoxicity against HepG2 (IC_50_ 1.0 μg/mL) [24]. Compounds **44** and **45** were found to be inhibitors of cytochrome P_450_ 1A activity [25]. Compound **47** exhibits cytotoxic activity against HepG2, HCT-116, and HeLa cells with low IC_50_ μg/mL values [26]. Cytotoxic activity against human hepatocarcinoma (HepG2) and breast adenocarcinoma (MCF-7) cell lines was observed for **48** and **49** [21].

Compounds **66** and **68** have significant cytotoxic activity against the human hepatocellular liver carcinoma cell line HepG2 with an IC_50_ of 20 μM while **67** and **68** show activity against the human breast adenocarcinoma cell line MCF-7, also with an IC_50_ in the low μM range. The anti-proliferative activity of **66** and **68** can be attributed, at least in part, to observed cellular apoptosis activity [13,30]. Compound **70** exhibits cytotoxicity to a variety of cell lines including murine leukemia (P-388), human lung carcinoma (A-549), human colon carcinoma (HT-29) and human melanoma (MEL-28) [31].

**Table 3:** Cembrane diterpenes, sources and activities.

No.	Name	Source	Activity
29	Cembrene-C [12]	*Sarcophyton trocheliophorum*	anti-fungal, anti-bacterial
30	Sarcophine [19,22]	*S. glaucum*	anti-tumor, antifungal
31	(+)-7α,8β-Dihydroxydeepoxy-sarcophine [22]	*S. glaucum*	
32	Sarcophytolide 1 [19,30]	*S. glaucum*	anti-tumor
33	(1*S*,2*E*,4*R*,7*E*,11*E*,13*S*)-Cembratrien-4,13-diol [22]	*S. glaucum*	anti-tumor
34	(1*S*,2*E*,4*R*,6*E*,8*R*,11*S*,12*R*)-8,12-Epoxy-2,6-cembradiene-4,11-diol [22]	*S. glaucum*	anti-tumor
35	(1*S*,2*E*,4*R*,6*E*,8*S*,11*R*,12*S*)-8,11-Epoxy-4,12-epoxy-2,6-cembradiene [22]	*S. glaucum*	anti-tumor
36	Trochelioid A [23]	*S. trocheliophorum*	
37	Trochelioid B [23]	*S. trocheliophorum*	
38	16-Oxosarcophytonin E [23]	*S. trocheliophorum*	
39	*ent*-Sarcophine [23]	*S. trocheliophorum*	
40	8-*epi*-Sarcophinone [23]	*S. trocheliophorum*	
41	Sarcotrocheliol acetate [12]	*S. trocheliophorum*	anti-tumor
42	Sarcotrocheliol [12]	*S. trocheliophorum*	anti-tumor
43	Durumolide C [24]	*Sinularia polydactyla*	anti-fungal, anti-bacterial
44	11(*S*)-Hydroperoxylsarcoph-12(20)-ene [22]	*S. glaucum*	anti-fungal, anti-bacterial
45	12(*S*)-Hydroperoxylsarcoph-10-ene [25]	*S. glaucum*	cytotoxic HepG2 (anti-tumor)
46	(2*R*,7*R*,8*R*)-Dihydroxy-deepoxysarcophine [26]	*S. glaucum*	anti-tumor
47	7β-Acetoxy-8α-hydroxy-deepoxysarcophine [26]	*S. glaucum*	cytotoxic (HepG2)( anti-tumor)
48	7-Keto-8α-hydroxy-deepoxysarcophine [21]	*S. ehrenbergi*	cytotoxic (HepG2) (anti-tumor)
49	7β-Chloro-8α-hydroxy-12-acetoxy-deepoxysarcophine [21]	*S. ehrenbergi*	cytotoxic (HepG2) (anti-tumor)
50	Nephthenol [27]	*Lobophytum pauciflorum*	
51	Cembrene-A [27]	*Alcyonium utinomii*	
52	Alcyonol A [27]	*A. utinomii*	
53	Alcyonol B [27]	*A. utinomii*	
54	Alcyonol C [27]	*A. utinomii*	
55	Pauciflorol A [27]	*L. pauciflorum*	
56	Pauciflorol B [27]	*L. pauciflorum*	
57	Thunbergol [27]	*L. pauciflorum*	
58	Labolide [27]	*L. crassum*	
59	20-Acetylsinularolide B [27]	*L. crassum*	
60	20-Acetylsinularolide C [27]	*L. crassum*	
61	Sinularolide C [27]	*L. crassum*	
62	Sinularolide C diacetate [27]	*L. crassum*	
63	3-Deoxypresinularolide B [27]	*L. crassum*	
64	3-Deoxy-20-acetylpresinularolide B [27]	*L. crassum*	
65	Sarcophytol M [11]	*Litophyton arboreum*	
66	Sarcophytolol [13]	*Sarcophyton glaucum*	cytotoxic HepG2 (anti-tumor) antiproliferative
67	Sarcophytolide B [13]	*S. glaucum*	
68	Sarcophytolide C [13]	*S. glaucum*	
69	Deoxosarcophine [13]	*S. glaucum*	cytotoxic against MCF-7 (anti-tumor)
70	2-*epi*-Sarcophine [31]	*S. auritum*	cytotoxic
71	(1*R*,2*E*,4*S*,6*E*,8*R*,11*R*,12*R*)-2,6-cembradiene-4,8,11,12-tetrol [31]	*S. auritum*	cytotoxic
72	Singardin [31]	*Sinularia gardineri*	anti-tumor

### 3.2. Xenicane Diterpenes

**Figure 7:**
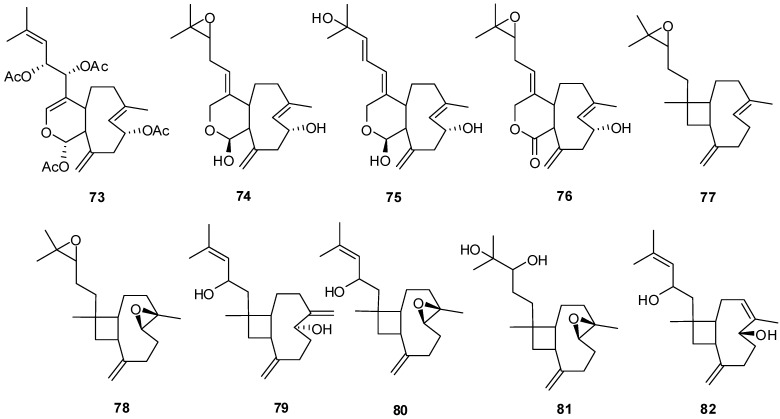
The structures of *Xenicane* diterpenes (**73**–**82**).

Bicyclo-[6,9]/[4,9]-diterpenes have been isolated from the coral genus *Xenia* with examples shown in Table 4 and Figure 7.

**Table 4:** Xenia diterpenes, sources and activities.

No.	Name	Source
73	Xenicin [28]	*Xenia macrosoiculata*
74	Xenialactol-D [28]	*X. obscuronata*
75	Xenialactol-C [28]	*X. obscuronata*
76	Xeniolide-E [28]	*X. obscuronata*
77	14(15)-Epoxyxeniaphyllene [28]	*X. lilielae*
78	Xeniaphyllene-dioxide [28]	*X. lilielae*
79	Xeniaphyllenol-C [28]	*X. macrosoiculata*
80	Epoxyxeniaphyllenol-A [28]	*X. lilielae*, *X. macrosoiculata*
81	l4,15-Xeniaphyllandiol-4,5-epoxide [28]	*X. macrosoiculata*
82	Xeniaphyllenol-B [28]	*X. macrosoiculata*

### 3.3. Eunicellin-Based Diterpenes

**Figure 8:**
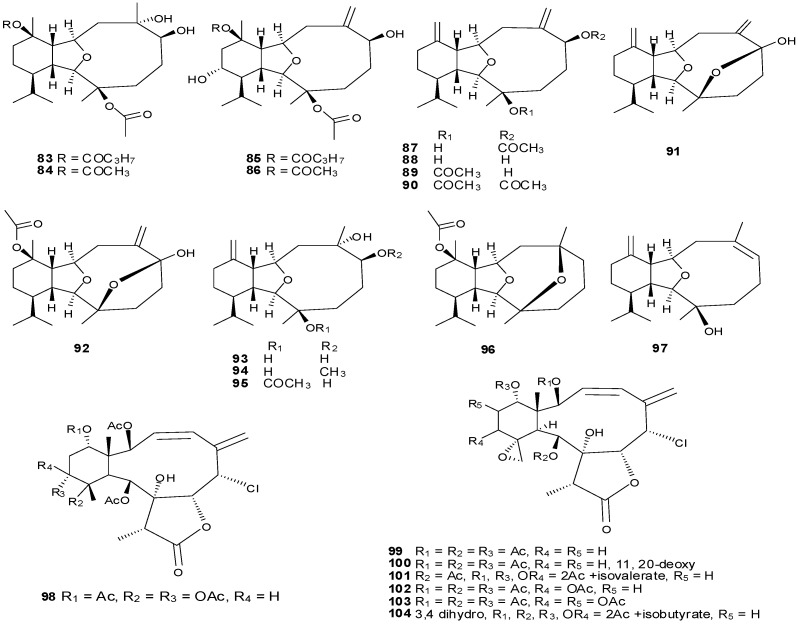
The structure of eunicellin-type diterpenes (**83**–**104**).

Tricyclo-[6,5,10]-diterpenes have been isolated from the soft coral genus *Cladiella* with examples shown in Table 5 and Figure 8. Eunicellin-based diterpenes display a wide range of bioactivities including anti-inflammatory and antitumor activities [26]. Compounds **83**–**104** have been evaluated for activity to inhibit growth, proliferation, invasion and migration of a prostate cancer cell line with potent anti-migratory and anti-invasive activities observed. Compounds with exomethylene functionalities at C-7 and C-11 demonstrate low anti-migratory activity, however replacement of the exomethylene moiety at C-7 with a quaternary oxygenated carbon, appreciatively increases the activity, as observed for compounds **93**–**94** and **96** [29].

### 3.4. Miscellaneous Diterpenes

**Figure 9:**
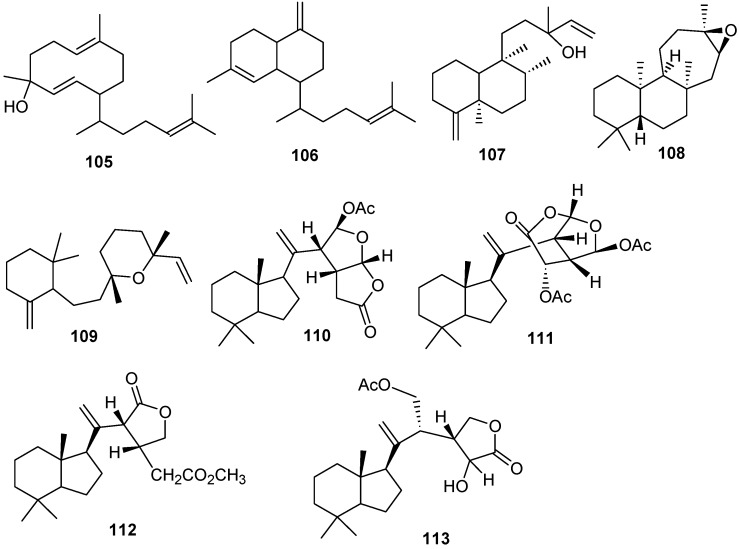
The structure of the macrocyclic type diterpenes (**105**–**113**).

Miscellaneous diterpenes were isolated from three different genus *Xenia*, *Chelonaplysilla* and *Dysidea*. These compounds were classified as: prenylated germacrenes (**105**), bicyclic diterpenes (**108**, **109**), clerodane diterpenes (**107**), carbo-tricyclic diterpenes (**108**) and re-arranged spongian diterpenes (**110**–**113**) as shown in Table 6 and Figure 9.

**Table 5:** Eunicellin diterpenoids, sources and activities.

No.	Name	Source	Activity
83	Pachycladin A [29]	*Cladiella pachyclados*	anti-tumor, anti-invasive
84	Klysimplexin G [29]	*C. pachyclados*	anti-tumor, anti-invasive
85	Pachycladin B [29]	*C. pachyclados*	anti-tumor, anti-invasive
86	Klysimplexin E [29]	*C. pachyclados*	anti-tumor, anti-invasive
87	Pachycladin C [29]	*C. pachyclados*	anti-tumor, anti-invasive
88	Cladiellisin [29]	*C. pachyclados*	anti-tumor, anti-invasive
89	3-Acetyl cladiellisin [29]	*C. pachyclados*	anti-tumor, anti-invasive
90	3,6-Diacetyl cladiellisin [29]	*C. pachyclados*	anti-tumor, anti-invasive
91	Pachycladin D [29]	*C. pachyclados*	anti-tumor, anti-invasive
92	Pachycladin E [26]	*C. pachyclados*	anti-tumor, anti-invasive
93	Sclerophytin A [29]	*C. pachyclados*	anti-tumor, anti-invasive
94	Sclerophytin F methyl ether [29]	*C. pachyclados*	anti-tumor, anti-invasive
95	Sclerophytin B [29]	*C. pachyclados*	anti-tumor, anti-invasive
96	(+)-Polyanthelin A [29]	*C. pachyclados*	anti-tumor, anti-invasive
97	Cladiella-6*Z*,11(17)-dien-3-ol [29]	*C. pachyclados*	anti-tumor, anti-invasive
98	Briarein A [32]	*Junceella juncea*	
99	Juncins A [32]	*J. juncea*	
100	Juncins B [32]	*J. juncea*	
101	Juncins C [32]	*J. juncea*	
102	Juncins D [32]	*J. juncea*	
103	Juncins E [32]	*J. juncea*	
104	Juncins [32]	*J. juncea*	

**Table 6:** Macrocyclic diterpenes, sources and activities.

No.	Name	Source
105	Obscuronatin [28]	*Xenia obscuronata*
106	Biflora-4,10(19),15-triene [28,33]	*X. obscuronata*
107	Chelodane [34]	*Chelonaplysilla erecta*
108	Barekoxide [34]	*C. erecta*
109	Zaatirin [34]	*C. erecta*
110	Norrisolide [35]	*Dysidea* sp.
111	Norrlandin [35]	*Dysidea* sp.
112	Seco-norrlandin B [35]	*Dysidea* sp.
113	Seco-norrlandin C [35]	*Dysidea* sp.

## 4. Sesterterpenes and Norsesterterpenes

### 4.1. Sesterterpenes

**Figure 10:**
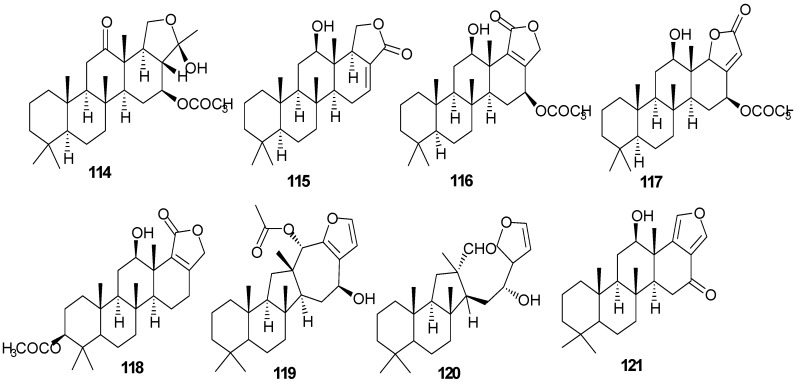

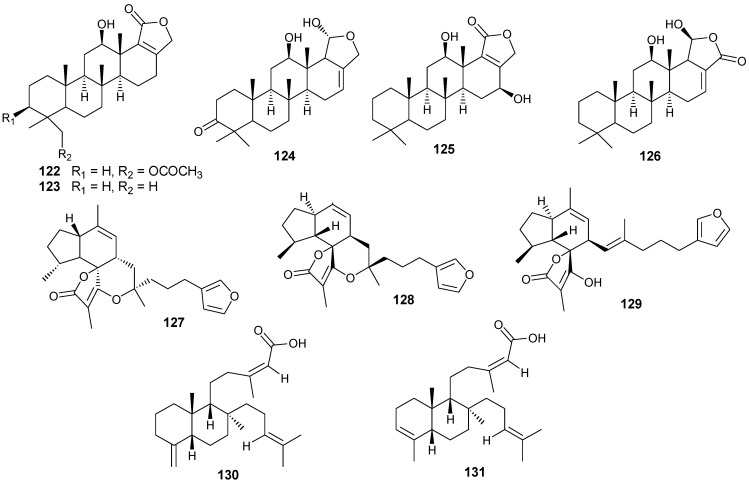
Structures of sesterterpenes (**114**–**131**).

Pentacyclo-[6,6,6,6,5]-sesterterpenes have been isolated from two different sponges with examples shown in Table 7 and Figure 10. Compound **116** exhibits antimycobacterial inhibition against *Mycobacterium tuberculosis* (H37Rv) at a concentration of 6 μg/mL while **117**–**119** displayed moderate to weak inhibitory activity [36]. Compounds **122**–**123** showed significant cytotoxicity against murine leukemia (P-388), human lung carcinoma (A-549) and a human colon carcinoma (HT-29) [37].

**Table 7:** Sesterterpenes, sources and activities.

No.	Name	Source	Activity
114	Scalardysin [18]	*Dysidea herbacea*	
115	25-Dehydroxy-12- *epi*-deacetylscalarin [36]	*Hyrtios erecta*	antimycobacterial
116	Sesterstatin [36]	*H. erecta*	antimycobacterial
117	16-*epi*-Scalarolbutenolide [36]	*H. erecta*	antimycobacterial
118	3-Acetylsesterstatin [36]	*H. erecta*	antimycobacterial
119	Salmahyrtisol A [37]	*H. erecta*	
120	Hyrtiosal [37]	*H. erecta*	
121	Salmahyrtisol B [37]	*H. erecta*	cytotoxic (anti-tumor)
122	19-Acetyl sesterstatin [37]	*H. erecta*	cytotoxic (anti-tumor)
123	Scalarolide [37]	*H. erecta*	
124	Salmahyrtisol C [37]	*H. erecta*	
125	16-Hydroxyscalarolide [38]	*H. erecta*	Cytotoxic, antimycobacterial
126	12-*O*-Deacetyl-12-*epi*-scalarine [38]	*H. erecta*	Cytotoxic, antimycobacterial
127	(−)-Wistarin [39]	*Ircinia wistarii*	
128	(+)-Wistarin [39]	*I. wistarii*	
129	(−)-Ircinianin [39]	*I.* *wistarii*	
130	Bilosespens A [40]	*Dysidea cinerea*	Cytotoxic
131	Bilosespens A [40]	*D. cinerea*	Cytotoxic

### 4.2. Norsesterterpenes

**Figure 11:**
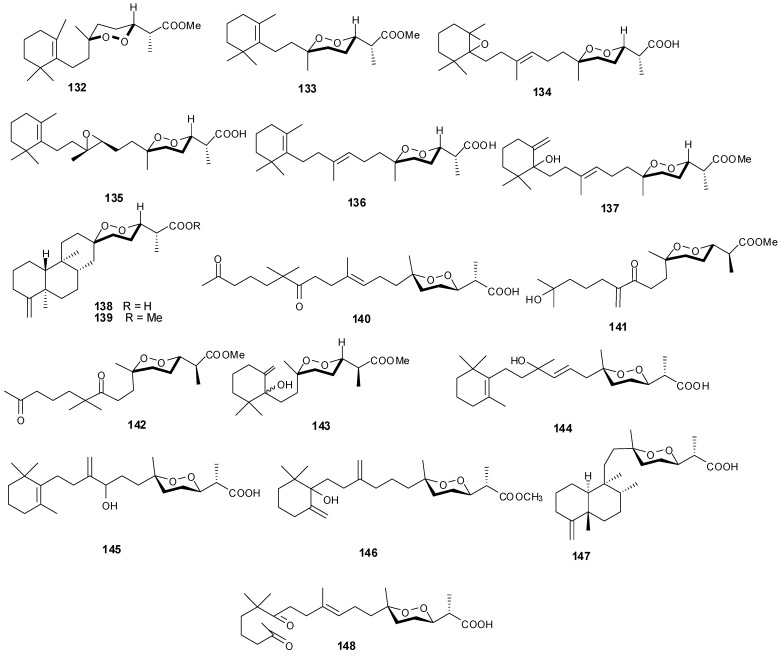
Structures of norterpenes (**132**–**148**).

Norsesterterpenes have been isolated from the sponge species *Diacarnus erythraeanus* with examples shown in Table 8 and Figure 11. Antitumor natural peroxide products are known to induce cytotoxicity in cancer cells through the generation of particular reactive oxygen species (ROSs). Compounds **134**–**135** displayed mean IC_50_ growth inhibitions less than 10 μM with several tumor cell lines [41]. However, additional studies with **135** established no *in vitro* selective growth inhibition between normal and tumor cells. In assaying three cancer cells including murine leukemia (P-388), human lung carcinoma (A-549) and human colon carcinoma (HT-29), **140**–**143** exhibited an IC_50_ greater than 1 μg/mL [15] while **145** showed lower cytotoxicity against the same lines [42].

**Table 8:** Norsesterterpenes, sources and activities.

No.	Name	Source	Activity
132	Nuapapuin A methyl ester [41]	*Diacarnus erythraeanus*	
133	Methyl-2-epinuapapuanoate [41]	*D. erythraeanus*	
134	(−)-13,14-Epoxymuqubilin A [41]	*D. erythraeanus*	anti-tumor
135	(−)-9,10-Epoxymuqubilin A [41]	*D. erythraeanus*	anti-tumor
136	(−)-Muqubilin A [41,43]	*D. erythraeanus*	anti-tumor
137	Hurghaperoxide [41]	*D. erythraeanus*	
138	Sigmosceptrellin B [41]	*D. erythraeanus*	
139	Sigmosceptrellin B methyl ester [41]	*D. erythraeanus*	
140	Aikupikoxide A [15]	*D. erythraeanus*	cytotoxic
141	Aikupikoxide D [15]	*D. erythraeanus*	cytotoxic
142	Aikupikoxide C [15]	*D. erythraeanus*	cytotoxic
143	Aikupikoxide B [15]	*D. erythraeanus*	cytotoxic
144	Tasnemoxide A [42]	*D. erythraeanus*	cytotoxic (anti-tumor)
145	Tasnemoxide B [42]	*D. erythraeanus*	cytotoxic (anti-tumor)
146	Tasnemoxide C [42]	*D. erythraeanus*	cytotoxic (anti-tumor)
147	*epi*-Sigmosceptrellin B [44]	*D. erythraeanus*	
148	Muqubilone [45]	*D. erythraeanus*	antimalarial

## 5. Triterpenes

**Figure 12:**
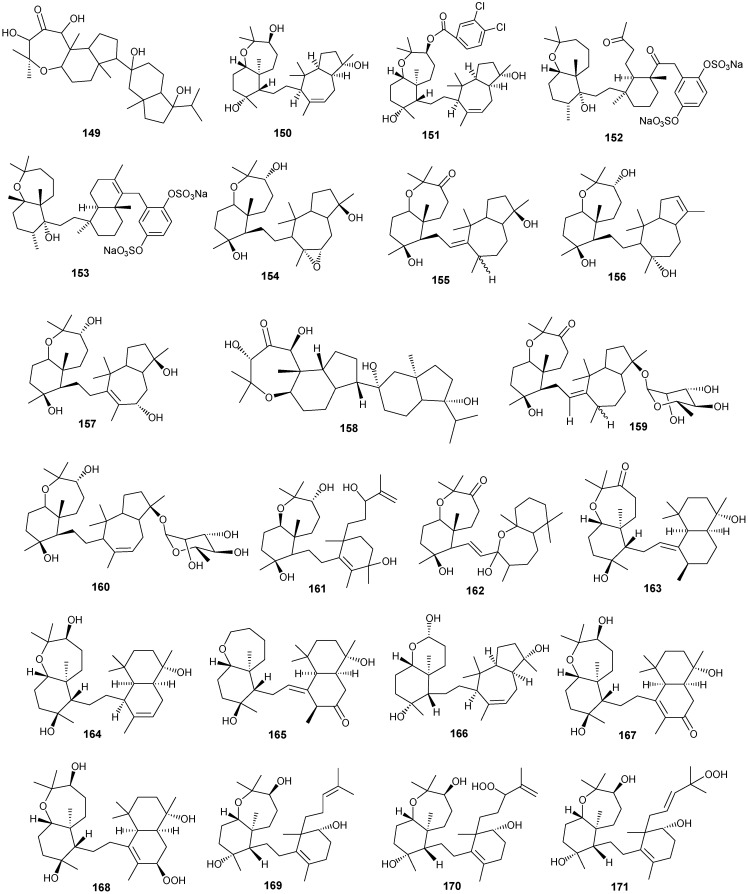

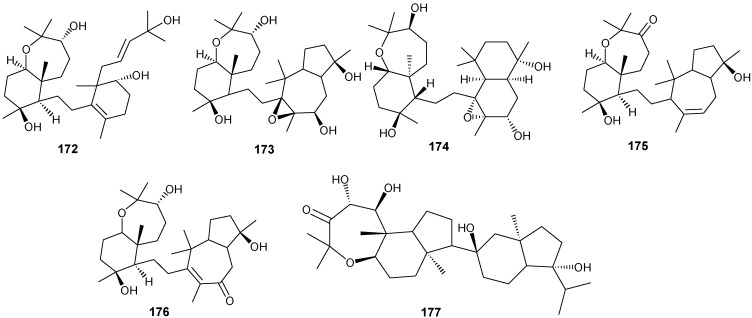
Structures of triterpenes (**149**–**177**).

Structurally diverse triterpenes are widespread in Red Sea sponges with examples shown in Table 9 and Figure 12. Compound **149** inhibits growth of human breast cancer cells, MDA-MB-231, MCF-7, BT-474 and T-47D, in a dose-dependent manner [46,47]. Triterpenes have also been studied for their efficacy in reducing the appearance of drug resistance. In the presence of many cytotoxic drugs, resistant cell variants appear, a process referred to as multidrug resistance (MDR). Overexpression of the ATP-binding cassette (ABC) transporter ABCB1/P-glycoprotein (P-gp) is one of the most common causes of MDR in cancer cells. P-gp a 170-kD transmembrane glycoprotein functions as a drug efflux pump that extrudes a wide spectrum of compounds including amphipathic and hydrophobic drugs. Sipholane triterpenoids can serve as P-gp inhibitors and are being developed to enhance the effect of chemotherapeutic drugs with MDR cancer cells *in vitro* and *in vivo* [33,36]. Compounds **162**–**163** enhanced cytotoxicity of several P-gp substrate-anticancer drugs, including colchicine, vinblastine and paclitaxel. These sipholane triterpenes significantly reversed the MDR-phenotype in P-gp-over expressing MDR cancer cells, KB-C2, in a dose-dependent manner. Moreover, these sipholanes have no effect on the response to cytotoxic agents in cells lacking P-gp expression or expressing MRP1 (ABCC1) or MRP7 (ABCC10) or with the breast cancer resistance protein (BCRP/ABCG2). Perhaps most importantly, these sipholanes with a low IC_50_ of *ca*. 50 μM are not toxic to the assayed cell lines [48].

**Table 9:** Triterpenes, sources and activities.

No.	Name	Source	Activity
149	Neviotine-A [46,47]	*Siphonochalina siphonella*	
150	Sipholenol A [47,49,50,51,52,53]	*S. siphonella*	anti-tumor
151	SipholenolA-4-*O*-3′,4′-dichlorobenzoate [49]	*S. siphonella*	
152	Shaagrockol B [54]	*Toxiclona toxius*	
153	Shaagrockol C [54]	*T. toxius*	
154	Sipholenol G [55]	*S. siphonella*	
155	Sipholenone D [55]	*S. siphonella*	
156	Sipholenol F [55]	*S. siphonella*	
157	Sipholenol H [55]	*S. siphonella*	
158	Neviotine B [55]	*S. siphonella*	
159	Sipholenoside A [55]	*S. siphonella*	
160	Sipholenoside B [55]	*S. siphonella*	
161	Siphonellinol B [55]	*S. siphonella*	
162	Dahabinone A [55]	*S. siphonella*	
163	Sipholenone E [51]	*S. siphonella*	anti-tumor
164	Sipholenol L [47,51]	*S. siphonella*	anti-tumor
165	Sipholenol J [51]	*S. siphonella*	
166	(2*S*,4a*S*,5*S*,6*R*,8a*S*)-5-(2-((1*S*,3a*S*,5*R*,8a*S*,*Z*)-1-hydroxy-1,4,4,6-tetramethyl-1,2,3,3a,4,5,8,8a-octahydroazulen-5-yl)-ethyl)-4a,6-dimethyloctahydro-2*H*-chromene-2,6-diol [51]	*S. siphonella*	
167	Sipholenol K [51]	*S. siphonella*	
168	Sipholenol M [51]	*S. siphonella*	
169	Siphonellinol D [51]	*S. siphonella*	
170	Siphonellinol E [51]	*S. siphonella*	
171	Siphonellinol-C-23-hydroperoxide [51]	*S. siphonella*	
172	Siphonellinol C [56]	*S. siphonella*	
173	*epi*-Sipholenol I [56]	*S. siphonella*	
174	Sipholenol I [51]	*S. siphonella*	
175	Sipholenone A [56,47]	*S. siphonella*	
176	Sipholenol D [52]	*S. siphonella*	
177	Neviotine-C [47]	*Siphonochalina siphonella*	cytotoxic

## 6. Steroids

**Figure 13:**
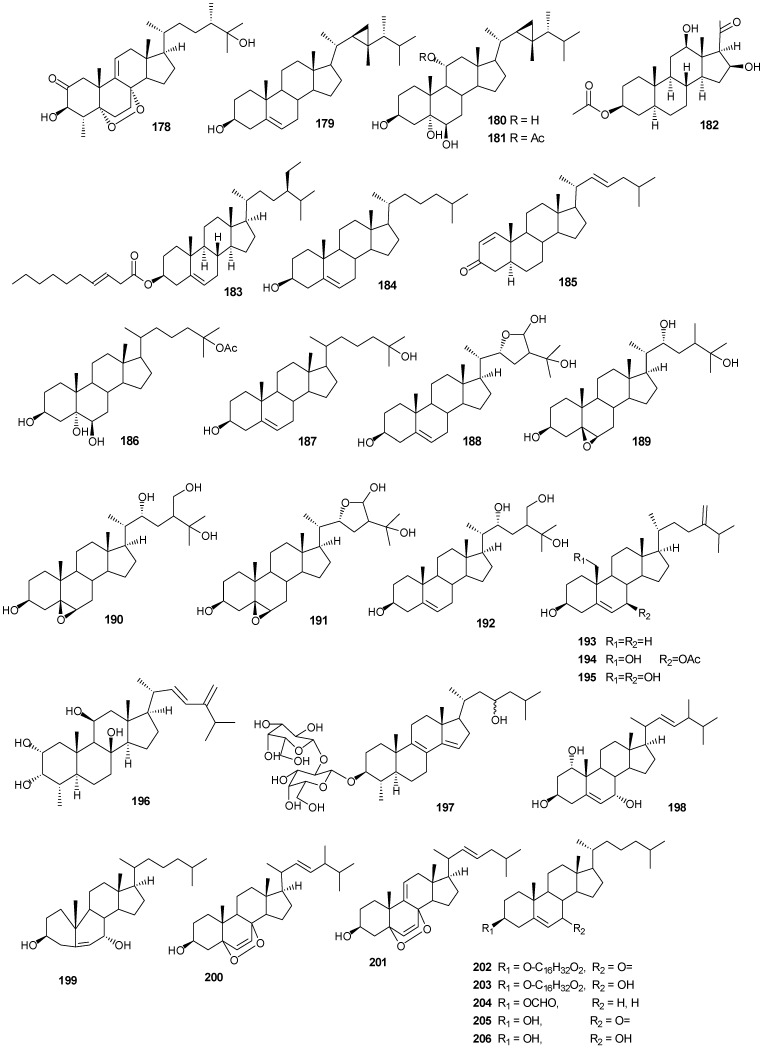

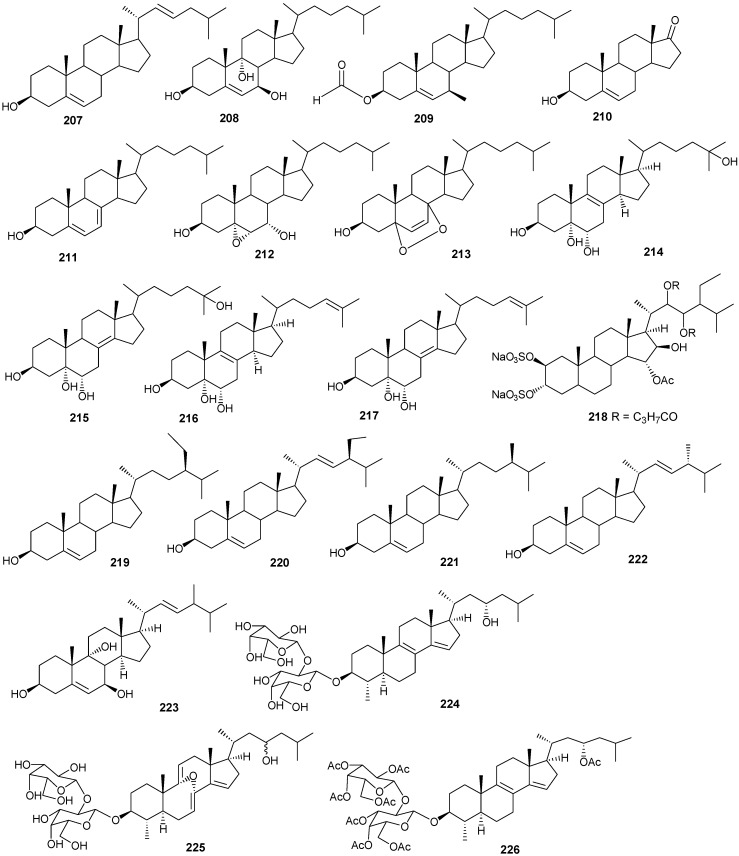
Steriod structures (**178**–**226**).

Steriods are widespread throughout the marine biome with recent chemical reports including soft coral (*Sinularia candidula*, *S. polydactyla*, *Heteroxenia ghardaqensis*, *Dendronephthya* sp*.*, *Lobophytom depressum* and *Litophyton arboreum*) [9,11,24,57,58,59,60], black coral (*Antipathes dichotoma*) [38,40], and sponges (*Echinoclathria gibbosa*, *Hyrtios* sp., *Erylus* sp., and *Petrosia* sp.) [18,64,65,66]. Steroid examples are shown in Table 10 and Figure 13.

**Table 10:** Steroids, sources and activities.

No.	Name	Source	Activity
178	3β-25-Dihydroxy-4-methyl-5α,8α-epidioxy-2-ketoergost-9-ene [57]	*Sinularia candidula*	anti-viral
179	Gorgosten-5(*E*)-3β-ol [58]	*Heteroxenia ghardaqensis*	anti-tumor
180	Gorgostan-3β,5α,6β,11α-tetraol (sarcoaldosterol A) [58]	*H. ghardaqensis*	
181	Gorgostan-3β,5α,6β-triol-11α-acetate [58]	*H. ghardaqensis*	
182	5α-Pregna-3β-acetoxy-12β,16β-diol-20-one [59]	*Echinoclathria gibbosa*	anti-tumor
183	β-Sitosterol-3-*O*-(3*Z*)-pentacosenoate [59]	*E. gibbosa*	anti-tumor
184	Cholesterol [9]	*Dendronephthya*	
185	Dendronesterone A [9]	*Dendronephthya*	
186	24-Methylcholestane-3β,5α,6β,25-tetrol-25-monoacetate [24]	*Sinularia polydactyla*	anti-tumor
187	24-Methylcholestane-5-en-3β,25-diol [24]	*S. polydactyla*	antimicrobial
188	Lobophytosterol [60]	*L. depressum*	
189	5β,6β-Epoxy-24*E*-methylchloestan-3β,22(*R*),25-triol [60]	*L. depressum*	
190	Depresosterol [60]	*L. depressum*	
191	(22*R*,24*E*,28*E*)-5β,6β-Epoxy-22,28-oxido-24-methyl-5α-cholestan-3β,25,28-triol [60]	*L. depressum*	
192	(22*R*,24*E*)-24-Methylcholest-5-en-3β,22,25,28-tetraol [60]	*L. depressum*	
193	24-Methylcholesta-5,24(28)-diene-3β-ol [11]	*Litophyton arboreum*	
194	7β-Acetoxy-24-methylcholesta-5-24(28)-diene-3,19-diol [11]	*L. arboreum*	cytotoxic
195	24-Methylcholesta-5,24(28)-diene-3β,7β,19-triol [11]	*L. arboreum*	
196	Hyrtiosterol [16]	*Hyrtios* Species	
197	Eryloside A [61,62,63]	Genus *Erylus*	cytotoxic
198	(22*E*)-Methylcholesta-5,22-diene-1α,3β,7α-triol [64]	*Antipathes dichotoma*	anti-bacterial
199	3β,7α-Dihydroxy-cholest-5-ene [64]	*A. dichotoma*	anti-bacterial
200	(22*E*,24*S*)-5α,8α-Epidioxy-24 methylcholesta -6,22-dien-3β-ol [64]	*A. dichotoma*	anti-bacterial
201	(22*E*,24*S*)-5α,8α-Epidioxy-24-methylcholesta-6,9(11),22-trien-3β-ol [64]	*A. dichotoma*	anti-bacterial
202	3β-Hexadecanoylcholest-5-en-7-one [65]	*A. dichotoma*	anti-tumor
203	3β-Hexadecanoylcholest-5-en-7β-ol [65]	*A. dichotoma*	anti-tumor
204	Cholest-5-en-3β-yl-formate [65]	*A. dichotoma*	anti-tumor
205	3β-Hydroxycholest-5-en-7-one [65]	*A. dichotoma*	
206	Cholest-5-en-3β,7β-diol [65]	*A. dichotoma*	
207	22-Dehydrocholestrol [65]	*A. dichotoma*	
208	3β,7β,9α-Trihydroxycholest-5-en [66]	*Petrosia*	cytotoxic (anti-tumor)
209	Cholest-5-en-7β-methyl-3β-yl formate [66]	*Petrosia* sp*.*	cytotoxic (anti-tumor)
210	Dehydroepiandrosterone [66]	*Petrosia* sp.	cytotoxic (anti-tumor)
211	7-Dehydrocholesterol [66]	*Petrosia* sp.	cytotoxic (anti-tumor)
212	5α,6α*-*Epoxycholest-8(14)-ene-3β,7α-diol [66]	*Petrosia* sp.	cytotoxic (anti-tumor)
213	5α,8α-Epidioxycholesta-6-en-3β-ol [66]	*Petrosia* sp.	cytotoxic (anti-tumor)
214	Cholesta-8-en-3β,5α,6α,25-tetrol [67]	*Lamellodysidea herbacea*	
215	Cholesta-8(14)-en-3β,5α,6α,25-tetrol [67]	*L. herbacea*	
216	Cholesta-8,24-dien-3β,5α,6α-triol [67]	*L. herbacea*	anti-fungal
217	Cholesta-8(14),24-dien-3β,5α,6α-triol [67]	*L. herbacea*	anti-fungal
218	Clathsterol [68]	*Clathria* sp.	
219	Clionasterol [69]	*Dragmacidon coccinea*	
220	Stigmasterol [69]	*D. coccinea*	
221	Campesterol [69]	*D. coccinea*	
222	Brassicasterol [69]	*D. coccinea*	
223	Dendrotriol [70]	*Dendronephthya hemprichi*	
224	Erylosides K [62]	*Erylus lendenfeldi*	
225	Erylosides L [62]	*E. lendenfeldi*	
226	Erylosides B [63]	*E. lendenfeldi*	

Moderate growth inhibition for a human colon tumor cell line was observed with **180** [58]. Compounds **184** and **186** exhibited activity against three human tumor cell lines including the lung non-small cell line A549, the glioblastoma line U373 and the prostate line PC-3 [59]. Compound **187** showed an IC_50_ of 6.1 and 8.2 μg/mL against the human cancer cell lines HepG2 and HCT, respectively [24].

Compounds **204**–**207** show antibacterial activity against Gram-positive (*Bacillus subtilis*) and Gram-negative bacteria (*Pseudomonas aeruginosa*), at a 1 μg/ml concentration [64]. Compounds **208**–**210** exhibited antitumor activity based on four cancer panels: HepG2, WI 38, VERO, and MCF-7 [65]. Compounds **215**–**220** exhibited cytotoxic effects in the tumor cell lines, HepG2 and MCF-7 with IC_50_ in the range of 20-500 μM. Interestingly, **217** showed the highest affinity to DNA with IC_50_ 30 μg/mL [66]. Compounds **223** and **224** showed antifungal activity against *Candida tropicalis*, with petri dish inhibition at 10 μg/disc [67].

## 7. Drug Leads

Even though terpenes are the largest group of natural products with over 25,000 structures thus far reported, a small subset of these metabolites have been investigated for biological function and/or activity. Basic biological constituents such as membrane components, hormones, antioxidants and chemical defenses require the isoprenoid building module. Future chemical studies of marine organisms are expected to generate an ensemble of novel terpenes based on progressive knowledge on enzymatic machinery and selective pressures under which such organisms have evolved. The expanding chemo-diversity of marine terpenes is being assisted in part by advanced analytical chemistry methods for structure determination and sophisticated diving techniques for sample collection.

Methods for assaying for *in vitro* biological activity can be more variable in terms of stardardized protocols. The same positive or native chemical controls are not always utilized making direct comparisons of biological activity between different testing laboratories unreliable or at least not reproducible. Moreover, with the paucity of ethnomedical knowledge from marine sources, the basis for selecting the most promising bioassay can be more of an art than a science. The screening for anti-cancer activity in facilities such as the National Cancer Institute (NCI) Chemotherapeutic Agents Repository operated by Fisher BioServices [71] can provide invaluable, cost-free, sensitive screening of hits against multiple-target 60 cancer cell line panels, broadening the opportunity to conduct more comprehensive and mechanistic studies. In the case where a set of metabolites has already been identified possessing a given biological activity, computational, *in silico*, and pharmacophore modeling can guide future design of druggable analogues with better biological activity, without expected toxicity, even if the structural characterization of the biological target(s) is/are not feasible. Such virtual models utilizes steric and electronic descriptors to identify pharmacophoric features such as hydrophobic centroids, aromatic rings, hydrogen bonding acceptors/donors and cation/anion interactions to match optimal supramolecular interactions with a specific biological target that triggers or blocks a response. Functional group properties can also be identified for the rational semi-synthetic design of biologically active marine natural scaffolds. Strategies such as the Topliss scheme designate a series of substituents based on lipophilic, electronic and steric properties to generate multiple analogues with slight controlled chemical property differences that can be used for comprehensive structure-activity studies to obtain superior biological activity relative to the parent natural product. While these techniques and tools are not distinct or exclusive for exploring marine sources and marine-derived natural products, such methods can be effective for enhancing biological activity. For example, sipholenol A is a noteworthy example of developing a marine metabolite using medicinal chemistry approaches to generate biologically active analogue libraries [49]. These natural product examples with exceptional biological potency outcomes (IC_50_ in the low μM range for invasive breast cancer) demonstrate the potential of marine natural products for the discovery of future novel druggable entities useful for the control and management of human diseases.

## 8. Conclusions

Terpenoids provide a vast array of molecular architectures with the coral community of the Red Sea having added significantly to the structure database over the last thirty years. While marine invertebrates in this ecosystem are still being discovered, interest in both the chemistry and biological activity of Red Sea terpenes has generated many novel structures with promising biological activities.
